# Relation between bilateral differences in internal jugular vein caliber and flow patterns of dural venous sinuses

**DOI:** 10.1007/s12565-013-0176-z

**Published:** 2013-04-10

**Authors:** Kazunobu Saiki, Toshiyuki Tsurumoto, Keishi Okamoto, Tetsuaki Wakebe

**Affiliations:** Unit of Translational Medical Science, Department of Macroscopic Morphology, Nagasaki University, Graduate School of Biomedical Science, 1-12-4 Sakamoto, Nagasaki, 852-8523 Japan

**Keywords:** Internal jugular vein, Superior sagittal sinus, Transverse sinus, Groove for superior sagittal sinus, Bilateral differences

## Abstract

We measured the calibers of the left and right internal jugular veins (IJV) and sizes of the left and right transverse sinuses (TS) in 91 cadavers, compared them between the left and right sides, and also evaluated the drainage patterns of the superior sagittal sinus (SSS) and straight sinus (=rectal sinus, RS) in the torcular Herophili. In addition, the running type of groove for the SSS was investigated. The results were as follows: (1) The right IJV was larger in 81.3 %, while the left IJV was larger in only 11.0 %. (2) The drainage pattern of the SSS was the right type in 73.6 %, intermediate type in 14.3 %, and left type in 12.1 %. (3) The drainage pattern of the RS was the right type in 27.5 %, intermediate type in 25.3 %, and left type in 47.3 %. (4) The running type of groove for the SSS was mostly consistent with the drainage pattern of this sinus. (5) Concerning the relationships among these findings including the size of the TS, the drainage pattern of the SSS was mostly consistent with the side showing a larger TS as well as the side showing a larger IJV. These results suggest that the pattern of drainage of the SSS into the left and right TS affects the size of the TS and the running type of groove for the SSS, and is also closely involved in the caliber of the IJV. A discussion of the embryological, genetic, and clinical implications of these results is presented.

## Introduction

The internal jugular vein (IJV) is a major vein collecting blood from the head and neck and is also a clinically important vein. The right IJV is known empirically to be larger than the left IJV. In recent years, ultrasonographic studies on the differences in IJV caliber between the left and right sides have been performed (Matsuda et al. 2005). However, there have been few macroscopic anatomical studies (Goto and Koda 2000) and no studies in which measurements or statistical analysis were performed.

Goto and Koda (2000) described in their book that the right IJV is frequently larger than the left IJV, and the side showing a larger IJV is mostly consistent with the side showing a larger sigmoid sinus, suggesting a relationship between the dural sinus pathway and IJV caliber. Concerning the dural sinus pathway, an anatomy textbook mentions that the superior sagittal sinus (SSS) often drains into the right transverse sinus (TS), while the straight sinus (=rectal sinus, RS) tends to drain into the TS contralateral to SSS drainage (Standring 2005: Gray’s anatomy). Gibbs and Gibbs (1934), Woodhall (1936), Kaplan et al. (1972), and Goto and Koda (2000) evaluated the drainage patterns of these sinuses, and confirmed the above tendencies. In recent years, Singh et al. (2004) and Fukusumi et al. (2010) performed image reconstruction to evaluate flow in the SSS in the torcular Herophili region using CT and MRI, and observed similar tendencies. These findings suggest that the drainage pattern of the dural sinuses, particularly the SSS and RS, affects the IJV caliber.

To date, there have been no detailed studies investigating the drainage patterns of the SSS and RS, quantitative comparison of the sizes of the TS and IJV between the left and right sides, or the detailed evaluation of these relationships. The IJV and subclavian vein are the veins used most commonly for central venous catheterization, involving the risk of pneumothorax and injury of the thoracic duct or the right lymphatic duct due to puncture. In addition, thrombosis occasionally occurs in the dural sinuses, and its incidence is high in the SSS and TS (Ferro et al. 2004). Therefore, it is also clinically important to correctly understand the difference in IJV caliber between the left and right sides and the pathway of the dural sinuses.

In order to clarify the relationship between the SSS drainage pattern and sizes of the left and right TS as well as the calibers of the left and right IJVs, in this study, we measured IJV and TS sizes, and investigated the SSS and RS drainage patterns in the torcular Herophili. In addition, the relationship between the groove for the SSS and the groove for the TS in the internal occipital protuberance on the inner surface of the skull was also investigated.

## Materials and methods

We investigated 91 Japanese cadavers (47 males and 44 females; age range 35–98 years) used in autopsy training in the School of Medicine, Nagasaki University. All cadavers were supplied by body donation, and consent for their use for education and research had been obtained. Since no data allowing the identification of individuals were presented, there were no ethical problems in this study.

### Measurement of the IJV caliber

**Fig. 1:**
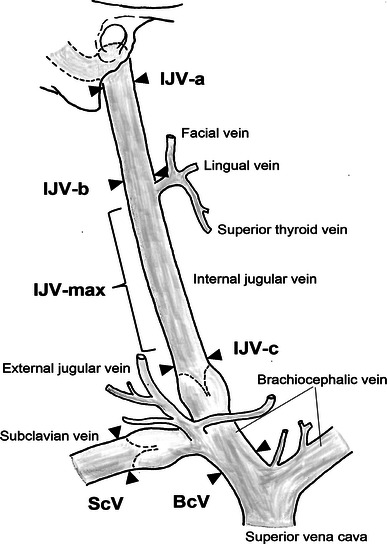
Measured points of the internal jugular, subclavian and brachiocephalic veins

The IJV caliber was measured at four sites: (1) immediately after exiting from the jugular foramen (IJV-a), (2) immediately above the site into which the facial vein and superior thyroid vein empty (IJV-b), (3) immediately above the inferior bulb of the IJV (IJV-c), and (4) the greatest site (IJV-max). In addition, the calibers of the subclavian vein (site about 2 cm distal to its confluence with the IJV) and brachiocephalic vein (the middle of the entire length) were measured as controls (Fig. 1). At each site, to exclude the influence of blood volume on the caliber, the blood vessel was pressed flat using tweezers, and the external diameter was measured using a vernier caliper (1-mm units).

### Measurement of the dural sinuses

**Fig. 2:**
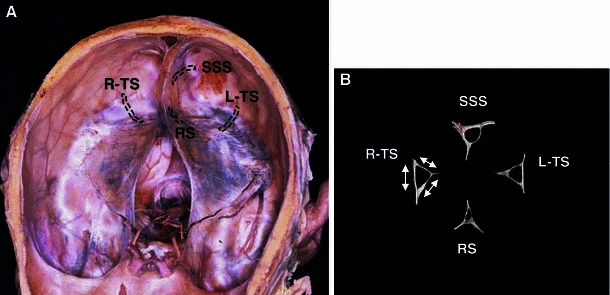
Measured points of the dural venous sinuses (**a**) and pieces crosscut off from the dural venous sinuses (**b**). The pieces were measured on three internal sides

For measurement of the dural sinuses, the Susa (1950) method was used. The SSS, right TS, and left TS were cut at a site about 3 cm distal to the center of the torcular Herophili, and the RS was cut at a site about 2 cm distal to it using a scalpel. The inner lengths of three sides were measured using a vernier caliper in 0.1-mm units (Fig. 2). The cross-sectional area was calculated from the lengths of the three sides using Heron’s formula. When the cross-sectional area was compared between the left TS and right TS in each individual, a difference ≥20 % of the larger cross-sectional area was considered to indicate the presence of a difference in size (R > L, R < L), and differences <20 % were considered to indicate equality in size between the left and right (R ≒ L).

### Classification of the drainage patterns of the SSS and RS

**Fig. 3:**
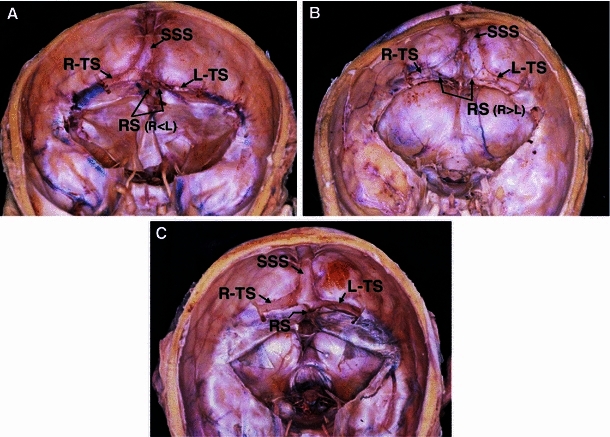
Three examples of opening the dural venous sinuses at the torcular Herophili. **a** The type of superior sagittal sinus (SSS) is perfect R; the type of the rectal sinus (RS) is imperfect L. *Arrows* in the RS indicate branches from the RS. **b** The SSS is imperfect L; the RS is imperfect R. **c** The SSS is RL (confluence); the RS is perfect L

**Fig. 4:**
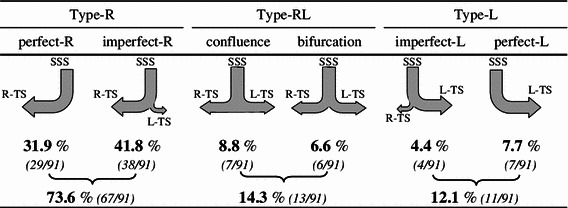
Classification of six flowing patterns in the SSS and their frequencies in the present study

**Fig. 5:**
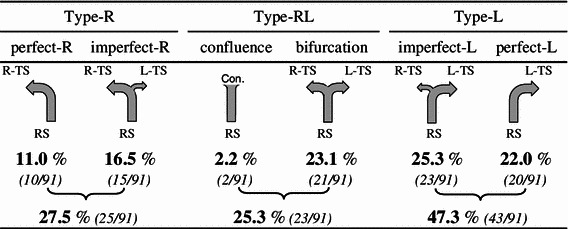
Classifications of six flowing patterns in the RS and their frequencies in the present study

Concerning the drainage patterns of the SSS and RS, the dural sinus was opened mainly in the torcular Herophili region (Fig. 3), and the drainage pattern was classified according to the classification of Goto and Koda (2000) into complete R, incomplete R, RL (intermediate), incomplete L, and complete L types. The RL type was subclassified into the confluence and bifurcation types (Figs. 4, 5).

### Classification of groove running type for the SSS on the inner surface of the skull

**Fig. 6:**
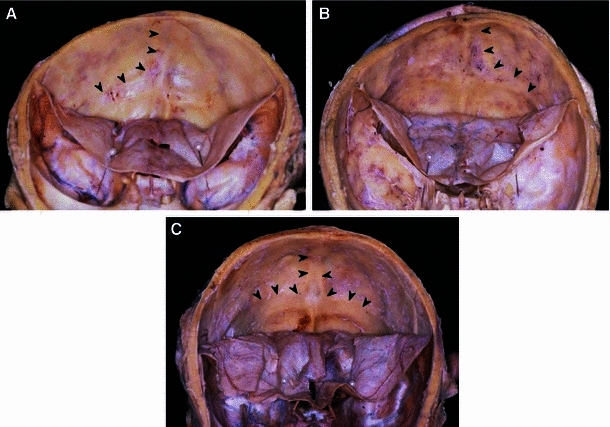
Three cases of running types of the groove for the SSS. **a** GS-R type, **b** GS-L type, **c** GS-RL type. The *arrows* indicate grooves for the SSS, and continuous groove for the TS

After dissection of the dura on the inner surface of the occipital bone, the running of the SSS groove was classified into the following three types (Fig. 6): groove continued to the right groove for the transverse sinus (GS-R), which continued to the left groove for the transverse sinus (GS-L), and then divided equally (GS-RL).

The mean caliber and cross-sectional area were analyzed using the paired *t* test, and categories were analyzed using the χ^2^ test by a computer software package from ‘Excel Statics version 5.0, 2002’ (Esumi, Tokyo, Japan).

## Results

### IJV caliber

**Fig. 7:**
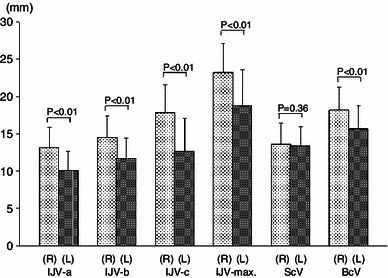
Comparison of the left and right caliber of the internal jugular vein (IJV), subclavian vein (ScV) and brachiocephalic vein (BcV). Except the ScV, the *right side* is significantly larger than the *left side*

The mean caliber of the right IJV was significantly greater (*P* < 0.01) at all sites (IJV-a, -b, -c, -max.) (Fig. 7). When the left and right IJV calibers were compared in each individual (IJV-c, differences ≥2 mm), the right IJV was larger in 81.3 %, and the left IJV was larger 11.0 %; the incidence differed markedly (Table 1). These incidences were relatively similar to those (R > L, 67.4 %; R < L, 12.1 %) reported by Goto and Koda (2000). The brachiocephalic vein showed a tendency similar to that of the IJV. The mean caliber of the subclavian vein was nearly equal between the left and right sides.

**Table 1:** Right and left side difference in caliber of the internal jugular (IJV), subclavian (ScV) and brachiocephalic (BcV) veins

	R > L	R ≒ L	L > R
IJV-c	81.3 % (74/91)	7.7 % (7/91)	11.0 % (10/91)
ScV	25.3 % (23/91)	57.1 % (52/91)	17.6 % (16/91)
BcV	60.4 % (55/91)	24.2 % (22/91)	15.4 % (14/91)

### Measurement values of the dural sinuses and the size difference between the right TS and left TS

**Fig. 8:**
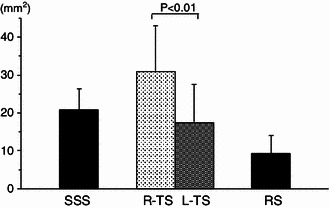
Comparison of average cross-sectional areas of the dural venous sinuses

The cross-sectional area was the largest for the right TS, followed in order by the SSS, left TS, and RS (Fig. 8). When the SSS and RS, which receive most blood in the cerebral hemispheres, were compared, the SSS showed a cross-sectional area twice that of the RS. When the left TS and right TS were compared, the mean cross-sectional area of R-TS was about 1.8 times that of left TS. In each individual, the right TS was larger in 71.4 %, and the left TS was larger only in 13.2 %. The results of this study were, on the whole, similar to those of previous studies (Table 2).

**Table 2:** Right and left side difference in size of the transverse sinus as shown in previous studies

	Total subjects	R > L	R ≒ L	R < L
Woodhall (1936)	100	39.0	48.0	13.0
Susa (1950)	120	68.0	16.0	16.0
Browning (1953)	100	51.0	20.0	29.0
Kaplan et al. (1972)	215	62.0	15.0	23.0
Ishizaka (1985)	52	52.0	19.2	28.8
Goto and Koda (2000)	132	63.6	22.7	13.6
Fukusumi et al. (2002)	200	68.5	10.0	21.5
Fukusumi et al. (2010)	120	63.3	21.7	15.0
Present study	91	71.4	15.4	13.2

### Relationship between SSS drainage pattern and TS size

**Fig. 9:**
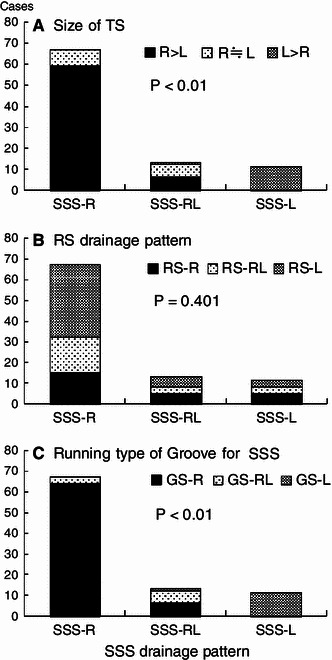
Relationship of the sizes of the left and right TS (**a**), the RS drainage patterns (**b**), and running types of the groove for the SSS (**c**) to the SSS drainage patterns. In the case of the SSS drainage R type, the right TS is mostly larger than the left TS (**a**), and running types of the groove for the SSS is mostly R type (**c**). Conversely, in the case of the SSS drainage L type, the left TS is larger (**a**), and running types of the groove for the SSS are also L type (**c**). There is no close relationship between SSS and RS drainage patterns (**b**)

The SSS drainage pattern in the torcular Herophili was the complete R type in 31.9 %, incomplete R type in 41.8 %, confluence type in 8.8 %, bifurcation type in 5.5 %, incomplete L type in 4.4 %, and complete L type in 7.7 %. In most cases, the SSS drained into the right TS (Fig. 4). Concerning the relationship between the SSS drainage pattern and TS size, the right TS size was larger in most cases showing the R type; there was no case showing left TS larger than right TS. In all cases showing the L type (complete and incomplete L types), the left TS was larger (Fig. 9a).

### Classification of the RS drainage pattern and its relationship with the SSS drainage pattern

The RS drainage pattern was classified as complete R type in 11.0 %, incomplete R type in 16.5 %, confluence type in 2.2 %, bifurcation type in 23.1 %, incomplete L type in 25.3 %, and complete L type in 22.0 % (Fig. 5). When the RS drainage pattern was classified into the R, RL, and L types, the incidences were 27.5, 25.3, and 47.3 %, respectively, showing a high incidence of the L type. Concerning the relationship between the RS and SSS drainage patterns, the R type of the SSS was observed more frequently in cases showing the L type of the RS, while the L type of the SSS was observed more frequently in cases showing the R type of the RS, although the differences were not significant (Fig. 9b).

### Relationship between the pattern of the groove in the internal occipital protuberance and the SSS

The GS-L type was observed in 12 cases, the GS-RL type in 9, and GS-R type in the other 70 (Fig. 9c). The groove pattern was mostly consistent with the SSS drainage pattern in both cases showing the GS-R type and those showing the GS-L type.

### Relationship between the TS size and IJV caliber

**Fig. 10:**
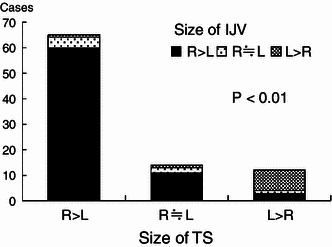
Relationship between the cross-sectional areas of the TS and the IJV calibers. Large sides of the TS accord with large sides of the IJV well

The right IJV tended to be larger in cases showing right TS larger than left TS, while the left IJV tended to be larger in cases showing left TS larger than right TS. In cases showing similar sizes of left TS and right TS, the right IJV tended to be larger. As a whole, the side showing a larger TS size was consistent with the side showing a larger IJV caliber (Fig. 10).

## Discussion

### SSS drainage pattern affects IJV caliber

Measurement of the sizes of the dural sinuses and evaluation of the drainage patterns of the SSS and RS showed that most or all SSS flow enters the right TS in most cases, as has been previously reported (e.g., Gibbs and Gibbs 1934; Woodhall 1936). Compared with the previous studies, the incidence of SSS draining into the right TS in our study was marked, while the SSS joining or bifurcating in the torcular Herophili and draining into the bilateral TSs was low (Table 3). In this study, the torcular Herophili region was cut and observed, and the sizes on the left and right sides at the bifurcation and flow due to the septum in the lumen were also taken into consideration for classification. Therefore, left or right predominance may have become clearer.

**Table 3:** Frequencies (%) of drainage patterns of continuity the SSS with the TS as shown in previous studies

	Total subjects	Type-R	Type-RL		Type-L	Other variations
			Confluence	Bifurcation		
Woodhall (1936)	100	30.0	9.0	52.0	9.0	―
Susa (1950)	120	52.6	10.8	25.8	10.8	―
Browning (1953)	100	18.0	36.0	40.0	6.0	―
Kaplan et al. (1972)	215	33	16	10	8	33
Ishizaka (1985)	52	17	37	23	15	8
Goto and Koda (2000)	131	52.7	31.3*		16.0	―
Fukusumi et al. (2002)	200	49	15	26	10	―
Singh et al. (2004)	160	41.0	35.0	14.0	10.0	―
Fukusumi et al. (2010)	120	44.2	20.8	26.6	9.2	―
Present study	91	73.6	8.8	6.6	12.1	―

* No distinction of ‘confluence’ and ‘bifurcation’

To evaluate the influences of the SSS drainage pattern on the TS and IJV sizes, their relationship in the order of venous flow is shown in Table 4. In cases showing SSS draining into the right TS, the right TS and IJV sizes were large. In cases showing SSS draining into the left TS, the left TS and IJV sizes were large. These results suggest that the SSS drainage pattern markedly affects IJV caliber.

**Table 4:**
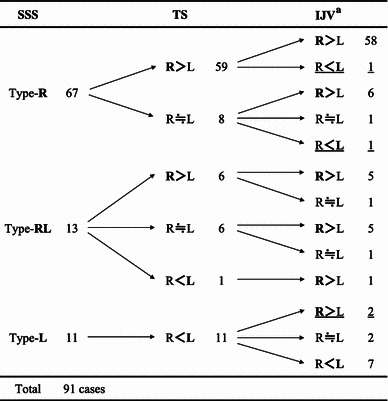
Relationship between drainage pattern of SSS, sectional sizes of TS, and IJV size

Although the SSS drainage pattern was consistent with the IJV caliber in most cases, a reverse relationship was observed in 5 of the 91 cases (Table 4). This suggests that the IJV caliber is also affected by draining veins after the TS (such as the superior and inferior petrosal sinuses and facial vein). In addition, certain diseases may also cause poor flow in the unilateral IJV and its surrounding veins, resulting in an increase in the contralateral IJV caliber.

The RS showed a slightly higher incidence of drainage into the left TS, although no significant difference was present. The RS drainage pattern may not have sufficient influence to determine the left–right difference in IJV caliber.

### Why does the SSS frequently drain into the right TS?: embryological discussion

**Fig. 11:**
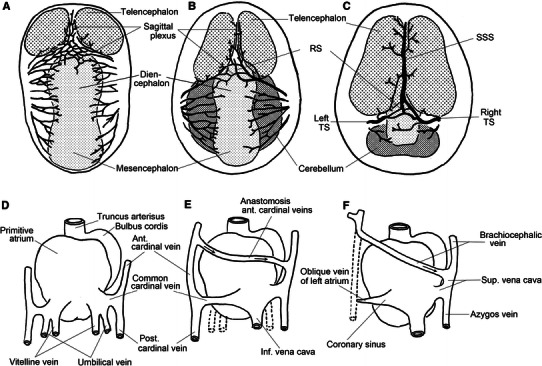
Schemes showing the development of the dural veins (**a**–**c**) and brachiocephalic vein (**d**–**f**). **a**–**c** Vertex view of the human embryo (quoted from Streeter 1915). **a** Embryo 13.8 mm long (6th week), **b** embryo 20 mm long (8th week); **c** embryo 54 mm long (9th–10th week). **d**–**f** Dorsal view of the heart and main veins (quoted from Moore 1988). **d** 4th week, **e** 7th week, **f** 8th week. Vein names in the adult are shown in **f**

Our results showed that the SSS frequently drains into the right TS, and, as a result, the right IJV becomes thicker. Why does the SSS frequently drain to the right? Since Padget (1957) and Masaki (1959) showed that the right TS tends to be larger in fetuses aged 4–10 months, the SSS drainage pattern may be determined at a relatively early stage of the prenatal period. Figure 11 shows a schematic diagram of the development of the dural sinuses and brachiocephalic vein, with reference to the reports of Streeter (1915) and Moore (1988). Based on these courses, the SSS, derived from the sagittal plexus, forms after the left anterior cardinal vein has anastomosed the right anterior cardinal vein. We speculate that the high incidence of SSS draining into the right TS is associated with the anastomosis of the left and right anterior cardinal veins (brachiocephalic veins later), in other words, disappearance of the left superior vena cava. Concerning the pathway from the dural sinuses to the heart through the IJV, the pathway through the right brachiocephalic vein is slightly shorter than that through the left brachiocephalic vein. The pathway through the right brachiocephalic vein shows linear flow into the heart, while the pathway through the left brachiocephalic vein causes a mild angle at the confluence. Thus, the vascular resistance is lower for drainage into the right TS than that into the left TS, and, therefore, the sagittal plexus flow may shift gradually to the right. Due to their relationships, the predominance of the right TS may increase, resulting in gradual increases in the right TS size and right IJV caliber.

To confirm this speculation, the absence of the predominance of the right IJV in cases of persistent left superior vena cava should be shown. However, this anomaly is very rarely observed, and time is necessary for the evaluation of substantial data. In addition, to clarify the embryological flow in the dural sinuses and IJV further, more detailed autopsy studies on the prenatal venous system are necessary.

### Relationship between SSS drainage pattern and groove around the internal occipital protuberance: genetics and group difference

In the internal occipital protuberance on the inner surface of the occipital bone, the SSS continues to the TS; a groove forms along the TS flow. However, there have been no studies in which the degree of consistency between the groove arrangement and sinus flow was confirmed. In this study, this consistency was also evaluated, and, as a result, the SSS drainage pattern was mostly consistent with the groove running type (Fig. 10).

In physical anthropology, the SSS groove that continues to the left TS groove is called the sagittal sinus groove to the left, and has frequently used been as a minor cranial variant (cranial non-metric variant) for the analysis of the lineage of races and groups (Dodo and Ishida 1990, Yamaguchi 1977, Saiki et al. 2000, Wakebe et al. 2012, etc.). The incidences of this variant in various groups, mainly in East Asia, are shown in Table 5. The incidence in each group is similar in the same region or period. Since the SSS drainage pattern is determined at an early stage of the prenatal period, this pattern and the groove formed based on this pattern may be affected more markedly by genetics than by the postnatal environment. The similar tendency in each region (Table 5) might be due to genetic influences. Although only the incidence of the sagittal sinus groove to the left has been evaluated in many previous studies, we classified the SSS groove arrangement into types, allowing application of the estimation of the SSS drainage pattern and the calibers of the left and right IJVs to ancient people.

**Table 5:** Incidences (%) of the sagittal sinus groove to the left in various populations in East Asia

Groups	Total subjects	Incidence (%)	Literature
Modern Japanese (Kanto and Tohoku)	153	17.0	Dodo and Ishida (1990)
Modern Japanese (Nagasaki)	227	18.1	Saiki et al. (2000)
Yayoi people (Northern Kyushu, Japan)	126	15.9	Dodo and Ishida (1992)
Yayoi people (Yamaguchi, Japan)	123	17.1	Dodo and Ishida (1988)
Yayoi people (Northwestern Kyushu, Japan)	76	7.9	Saiki et al. (2000)
Jomon people (Eastern Japan area, Japan)	127	11.8	Dodo and Ishida (1990)
Modern Korean	81	11.1	Takenaka (1994)
Modern Chinese (Shanxi Province)	30	13.3	Wang and Sun (1988)
Eastern Zhou dynasty age Chinese (Zhouzhuang)	116	12.1	Wakebe et al. (2011)
Eastern Zhou dynasty age Chinese (Xinghong)	63	14.3	Wakebe et al. (2011)
Modern Kazakh	116	20.7	Ishida (1995)
Iron age Tagar people	140	16.4	Ishida (1995)
Modern Japanese (Cadavers for the dissection course)	91	13.2	Present study

### Clinical importance

The right IJV is used most frequently as the site for central venous catheterization since the catheter can be advanced linearly to the superior vena cava, and the risk of pneumothorax as a complication is low. In general, the catheter is inserted into the right IJV for central venous catheterization (e.g., Yamayoshi 1996), but the right IJV is smaller than the left IJV in some patients, as was shown by the results of this study. This fact should be taken into consideration for this procedure. For accurate and safe central venous catheterization, the size and arrangement of the IJV should be confirmed using ultrasonography before puncture, as is recommended in the anesthesiological field (e.g., Karakitsos et al. 2006; Kunisawa 2009).

In the dural sinuses, thrombosis or stenosis sometimes occurs. Dural sinus occlusion due to thrombi is observed most frequently in the SSS, followed by the TS (Ferro et al. 2004; Saposnik et al. 2011). Multiple sinuses were reported to be involved in about one-third of patients (Bousser 1999). For the diagnosis of dural sinus thrombosis, CT and MRI have been used frequently in recent years. However, the interpretation of imaging findings is difficult, and misdiagnosis often occurs (Provenzale and Kranz 2011). Studies on the SSS drainage pattern in the torcular Herophili have shown individual differences and difficulty in determining whether the blood flow is original or due to thrombosis or occlusion when the flow is thin on the images obtained. In patients with dural sinus diseases such as sinus thrombosis, it should be taken into consideration that the drainage pattern of each sinus shows a certain tendency, but is not the same among patients, and appropriate diagnosis and treatment are necessary.

Moreover, Zamboni et al. (2006, 2009) recently proposed a new hypothesis for pathomechanisms of multiple sclerosis (MS): chronic cerebrospinal venous insufficiency (CCSVI). Research reports to verify this hypothesis have also increased (Doepp et al. 2010; Baracchini et al. 2011; Tanaka et al. 2011). The IJV and dural sinus are considerably associated with CCSVI. Although this study does not reveal the details of this relationship, we should conduct an investigation based on this hypothesis in future studies.

### Limitations

This study was performed in cadavers. After patient death, about 10 l preservative (containing formalin, ethanol, and water) was injected into the femoral and radial arteries. Therefore, the possibility that blood returned to the venous side, and the veins were dilated compared with the premortem state cannot be excluded. There is a possibility that the measurement results regarding veins in this study slightly differ from the premortem in vivo state.
